# Selected Species of the Cucurbitaceae Family Used in Mexico for the Treatment of Diabetes Mellitus

**DOI:** 10.3390/molecules27113440

**Published:** 2022-05-26

**Authors:** Maira Huerta-Reyes, Rosario Tavera-Hernández, J. Javier Alvarado-Sansininea, Manuel Jiménez-Estrada

**Affiliations:** 1 Unidad de Investigación Médica en Enfermedades Nefrológicas, Hospital de Especialidades “Dr. Bernardo Sepúlveda Gutiérrez”, Centro Médico Nacional Siglo XXI, Instituto Mexicano del Seguro Social, Cuauhtémoc, Ciudad de México 06720, Mexico; 2 Instituto de Química, Universidad Nacional Autónoma de México, Ciudad Universitaria, Coyoacán, Ciudad de México 04510, Mexico; rosario.tavera@gmail.com (R.T.-H.); manueljemex@gmail.com (M.J.-E.); 3 Herbario FEZA, Facultad de Estudios Superiores Zaragoza, Universidad Nacional Autónoma de México, Batalla de 5 de Mayo S/N, Col. Ejército de Oriente, Ciudad de México 09230, Mexico; javier33@comunidad.unam.mx

**Keywords:** Cucurbitaceae, Diabetes mellitus, hypoglycemic, antioxidant, anti-inflammatory, antidiabetic, α-glucosidase, α-amylase, traditional mexican medicine

## Abstract

In Mexico, Diabetes mellitus (DM) is a serious health problem, and although the current pharmacological treatments for DM such as insulin and oral hypoglycemics are available, the Mexican population continues to use medicinal plants in the treatment of DM. The antidiabetic properties of the plant species that belong to the Cucurbitaceae family has already been recognized worldwide. Since Mexico is one of the most important centers of diversity of Cucurbitaceae, the present work contributes to the review of the most used species of Cucurbitaceae in the treatment of DM in Mexico. The reviewed species (*Cucurbita ficifolia*, *C*. *maxima*, *C*. *moschata*, *C*. *pepo*, *Ibervillea* *sonorae*, *Sechium* *edule*, *Citrullus* *lanatus*, *Cucumis* *melo*, and *C*. *sativus*) revealed that the antidiabetic effects exerted are effective in a number of mechanisms involved in the complex pathogenesis of DM: hypoglycemic, antioxidant, anti-inflammatory, anti-obesity, protective effects on diverse organs and cells, as well as in the control of dyslipidemias; furthermore, the select species of the Cucurbitaceae family could also be essential components of diets for the control of DM in patients with the disease. Thus, the Cucurbitaceae species selected in the present work represent a source of antidiabetic agents that perhaps establish the bases for novel clinical treatments.

## 1. Introduction

Diabetes mellitus (DM) is a chronic disease that occurs when the pancreas is no longer able to produce insulin, or when the body cannot effectively use insulin, which causes an increase in blood glucose levels. Type 1 DM (T1DM) is caused by the loss of beta cells of pancreatic islets that produce insulin, resulting in the deficiency of insulin. In the case of Type 2 DM (T2DM), the disease is caused by insulin resistance and includes approximately 90% of all DM cases [1]. According to the International Diabetes Federation (IDF) [2], approximately 463 million persons (aged between 20 and 79 years) were living with DM worldwide in 2019, and by the year 2030, this number will rise to 700 million.

The guarantee of health services for covering the increasing demand by individuals affected by DM and associated complications represent a considerable economic burden with global impact [3]. In fact, a report on the year 2020 indicates that the persistent increasing global burden of DM varies by diabetic type, region, and country. Notoriously, in these rankings, Mexico is a country that appears in the top five places in the world: the highest prevalence of DM was registered in China (89.5 million), followed by India (67.8 million), the United States (30.7 million), Indonesia (21.0 million), and Mexico (13.1 million). Concerning countries by deaths caused by DM, the top places were occupied by India (254,555), China (153,185), Indonesia (97,005), the United States (68,558), and Mexico (64,067). Under the heading of disability-adjusted life-years associated with DM, the countries in the top were India (11.2 million), China (10.0 million), Indonesia (4.4 million), the United States (3.9 million), and Mexico (2.6 million) [4]. These data revealed the significance and proportions of DM in Mexico, where additionally, obesity, a risk condition closely related to DM, is increasing each year, especially in the child population [5]. As an effort in order to alert the general Mexican population, the Mexican Ministry of Health (SSA) declared DM as an epidemiological emergence in the country in the year 2016 [6].

Although in Mexico current pharmacological treatments for DM such as insulin and oral hypoglycemics are available, the Mexican population continues to use, and sometimes prefers, medicinal plants in the treatment of DM. Escandón-Rivera and coworkers in the year 2020 [7], reported that approximately 800 plants were being employed in Mexico for treating DM, either alone or in combination with drugs. Thus, the importance of the use of medicinal plants in Mexico reveals that the study of these species would be a rich source of possible active molecules that could offer a novel effect in the treatment of DM, and perhaps because they derive from plants, could have high acceptance for their use. Consequently, ethnobotanical information on the Mexican species utilized in the treatment of DM results is crucial.

The analysis reported by Hernández-Galicia and coworkers (2002) [8] disclosed valuable ethnobotanical knowledge for the control of DM in Mexico; this analysis was based on three different sources of information: (i) Bibliographic Investigation (mainly based on Mexican medicinal flora); (ii) Herbarium Research (Medicinal Plants Herbarium from the Instituto Mexicano del Seguro Social, IMSS-Herbarium); and (iii) the IMSS-COPLAMAR Program carried out between 1983 and 1985 (provided medical care and drugs to more than 13 million persons from rural and indigenous Mexican communities without Social Security in the country). This analysis also pointed out that the botanical families with the largest number of plant species used in Mexican traditional medicine for the control of DM are from the following families: Asteraceae, Leguminosae, Cactaceae, Euphorbiaceae, Labiatae, Rubiaceae, Solanaceae, Cucurbitaceae, and Rosaceae. Among these families, Cucurbitaceae is highlighted due to the high endemic number of the species in the country, the continuous common use by rural inhabitants as medicine, especially in DM treatment, and also because a number of Cucurbitaceae still thrive as wild species and as important crops as well, conferring on Cucurbitaceae significant importance as a natural source with international transcendence [9]. Therefore, this review provides a general and organized overview of the up-to-date knowledge of the most used Cucurbitaceae species that have been employed in Mexican traditional medicine for the treatment of DM. Since DM has no cure, the need to review the antidiabetic properties of the Cucurbitaceae species, as well as their phytochemical content, and the in vitro and in vivo assays employed, arises in order to contribute to the future development of novel and effective antidiabetic agents that perhaps will be complementary or alternative to those already used in clinical practice.

## 2. Generalities of the Cucurbitaceae Family

Cucurbitaceae is a flowering plant family of annual or perennial herbs or shrubs, also known as *cucurbits*. The leaves are alternate and exstipulate, while the flowers are unisexual, paniculate, racemose, or subumbellate. Calyx are mostly 5-lobed and imbricated and the corolla is valvate and involute. Seeds are numerous [10]. The fruit is perhaps the most notorious organ of the members of the Cucurbitaceae family and presents high variation in size, shape, and color. Mostly the fruits and, in some cases also the flowers, are eaten, and they are also used in traditional medicine and industry. Some fruits possess a bottle shape that has been utilized as a container or a musical instrument. Among the most recognized *cucurbits* worldwide are pumpkin, watermelon, melon, zucchini, and cucumber [11].

The Cucurbitaceae family together with the Asteraceae and Brassicaceae families has been considered extraordinarily important to humans due to their medicinal, alimentary, botanical, cultural, and economic relevance. Cucurbitaceae has been related with human nutrition for more than 12,000 years in that several species are among the plants first domesticated by humans [9,11]. Therefore, an interesting prevalent characteristic of Cucurbitaceae plants is their adaptability to a wide range of agricultural environments, because they grow as crops but can also be found in private gardens, with medicinal or food purposes [12]. Consequently, Cucurbitaceae species are cultivated around the world under a variety of conditions, highlighting their great economic importance, and they also have been considered the most diverse plant family on the planet [13].

The oils extracted from the seeds of Cucurbitaceae plants are using for cooking in Africa and the Middle East, and interest in its industrial applications has been increasing in recent years, due to the fact that their fatty acid and tocopherol compositions reveal a potential utilization in the industrial area as foods, detergents, vitamin supplements and biodiesel fuel [14].

The Cucurbitaceae family with a predominantly tropical distribution comprises approximately 118 genera and 825 species. Mexico is one of the most important centres of diversity of Cucurbitaceae, due to the 141 taxa that thrive in this country. From these latter, some estimations have calculated that 128 taxa thrive as wild and that 13 species are cultivated in Mexico. More than one half of these 128 Mexican wild taxa are endemic to this country and 34 are actively employed by people in rural areas at present [9,15].

Numerous species of Cucurbitaceae have been employed to cure a variety of diseases in different countries, in which the fruits and frequently the seeds are the most utilized parts of the plant. Information about Cucurbitaceae and its uses in traditional medicine are vast and has previously been the subject of many reports. In the present work, we mention some of the most frequent traditional uses of Cucurbitaceae reported worldwide. The treatment of digestive diseases is reported very often, such as intestinal parasites, constipation, flatulence, indigestion, colic, and stomachache [16,17,18]. To treat dermatological affections, Cucurbitaceae species were mentioned for dermatitis, acne, and dandruff [19,20,21]. Moreover, an immense variety of diseases such as hemorrhoids, fever, sore chest, paralysis, herpes, lung inflammation, asthma, malaria, leprosy, for blood cures, and headache are also treated with the Cucurbitaceae species [22,23,24]. A variety of investigations have demonstrated the biological properties of the members of the Cucurbitaceae family, such as antibacterial, antioxidant, anti-inflammatory, anticancer, antidiabetic, anti-HIV, anti-pyretic, and antihelminthic [13,25]. However, the traditional use of Cucurbitaceae in the treatment of chronic diseases such as DM, cancer, and arthritis is also documented [26,27,28]. Thus, the Cucurbitaceae family is a well-recognized source of secondary metabolites with diverse biological activities tested in in vitro and in vivo assays, and more recently, in clinical trials. Because of the extensive and complex information on the Cucurbitaceae species, the botanical significance of this plant family to Mexico, and the importance of DM in Mexico, the present manuscript focuses on the necessary information for the future study of bioactive molecules that are useful in the treatment of DM, those molecules deriving from the selected species of the Cucurbitaceae family most frequently reported as employed in Mexican traditional medicine for the treatment of DM as follows: *Cucurbita ficifolia*, *Cucurbita maxima*, *Cucurbita moschata, Cucurbita pepo*, *Ibervillea sonorae*, *Sechium edule*, *Citrullus lanatus, Cucumis sativus*, and *Cucumis melo*.

## 3. Selected Species of the Cucurbitaceae Family Used in Mexico in the Treatment of DM

### 3.1. Cucurbita ficifolia Bouché

*Cucurbita ficifolia* is one of the domesticated Cucurbitaceae species whose origin has not been defined. Some authors suggest Central America or Southern Mexico as places of origin, while others propose South America, and more specifically the Andes [29,30]. In Mexico, *C. ficifolia* is known as *chilacayote*, and is widely consumed in different traditional dishes and also as sweets prepared with their seeds or fruits [30]. The entire plant and the fruit are utilized in traditional medicine: for example, in the state of Hidalgo, Mexico, the fruit has been employed topically to treat worms found under the skin (such as *larva migrans*) [31], and the fruit macerated in water is used for the treatment of diabetes [30,32].

A number of in vitro and in vivo tests have been carried out to show the hypoglycemic effect of *C. ficifolia*. Román-Ramos et al. evaluated glucose tolerance in an animal model using healthy rabbits that were treated as follows: water as a control (4 mL/kg); Tolbutamide (20 mg/kg) as a reference control, and plant preparation (*C. ficifolia*, 4 mL/kg). *C. ficifolia* exhibited the greatest decrease in the area under the glucose tolerance curve (30.7%) [33]. Previous studies by the same research group showed that *C. ficifolia* possesses a hypoglycemic effect equal to that of Tolbutamide in healthy rabbits with temporary hyperglycemia (induced by subcutaneous administration of glucose) and in animals with moderate diabetes induced by Alloxan (fasting glucose level 150–300 mg/dL), but not in animals with severe diabetes induced by Alloxan (fasting glucose level higher than 400 mg/dL) [34]. In this model studied by Román-Ramos et al., Tolbutamide produces hypoglycemia in healthy animals, and in diabetic animals (T2DM) induced by Alloxan, which produces the release of insulin in the beta cells of the pancreas. In animals with severe Alloxan-induced diabetes (T1DM), pancreatic beta cells are not present; therefore, Tolbutamide does not produce a hypoglycemic effect. Based on the latter, it was proposed that the juice of the fruit of *C. ficifolia* acts only in the presence of insulin [34]. *C. ficifolia*, in addition to having a hypoglycemic effect in healthy rabbits and those with moderate diabetes, improves glucose tolerance in fasting healthy rabbits; thus, it is suggested that the integration of edible plants with hypoglycemic activity into the diet involves the possibility of a high effect of controlling and preventing DM.

On the other hand, a study with 10 patients with T2DM demonstrated that the administration of *C. ficifolia* juice, at a dose of 4 mL/kg of body weight, caused a decrease in blood glucose levels during 5 h after ingestion of the extract. These results are consistent with the hypoglycemic effect observed in studies of animals with moderately high glucose levels [35].

Other reports comprising in vivo tests indicated that the juice from the pulp of *C. ficifolia* was administered intraperitoneally and orally to healthy and Alloxan-induced diabetic mice. The juice administered intraperitoneally produced the greatest decrease in blood glucose in healthy and diabetic mice, even greater than Tolbutamide. Oral administration for 14 days at a dose of 1000 mg/kg of body weight per day to diabetic mice normalized glucose levels at the end of this period. At high doses (750–1250 mg/kg) administered intraperitoneally, the highest death rates of mice were recorded, opening the need for more detailed toxicological investigations [36].

Xia and Wang have conducted various investigations on the antihyperglycemic effect of the hydromethanolic extract (70%) of *C. ficifolia* administered in rats with diabetes induced by Streptozotocin, at a dose of 300 and 600 mg/kg of body weight per day for a 30 day period. These authors observed a significant reduction in blood glucose, glycosylated hemoglobin, and an increase in plasma insulin and total hemoglobin [37]. These same authors, in a subsequent investigation, analyzed the lipid profile and glucose tolerance of the same *C. ficifolia* extract, finding not only a hypoglycemic effect but also a significant decrease in triglycerides, low-density lipoproteins (LDL) and a significant increase in the high-density lipoprotein (HDL) level. They also reported a significant effect on glucose tolerance [38].

Bayat et al. [39] evaluated the effect of the *C. ficifolia* extract (100 g) and probiotic yogurt (150 g) alone or in combination, in a clinical trial in patients with T2DM during 8 weeks. The results showed that *C. ficifolia* extract and probiotic yogurt alone or in combination, has beneficial effects on lipid profile, glycemic control, and inflammation. Additionally, the administration of the combination of *C. ficifolia* extract and probiotic yogurt caused a notable decrease in atherogenic lipids and lipoproteins levels, and an increase in high-density lipoprotein cholesterol (HDL-C), suggesting a synergistic effect. However, more studies are necessary in order to determine the role of the parts of this possible synergistic effect. Finally, authors, suggested that the consumption of *C. ficifolia* and probiotic yogurt may help in the treatment of patients with diabetes.

In another study, the effect of an aqueous extract of *C. ficifolia* (200 mg/kg) on the enzymes involved in oxidative stress, glutathione disulfide (GSSG) and reduced glutathione (GSH), was investigated in mice with Streptozotocin-induced diabetes by oral administration, for 30 days. The aqueous extract of *C. ficifolia* in mice with diabetes significantly reduced glycemia, polydipsia, and hyperphagia and plasma lipid peroxidation. The GSH/GSSG ratio and its redox potential were restored, which explains part of the antioxidant properties of the extract. The authors support the use of the *C. ficifolia* extract as an alternative treatment for the control of DM, in addition to preventing complications caused by oxidative stress [40].

**Figure 1:**
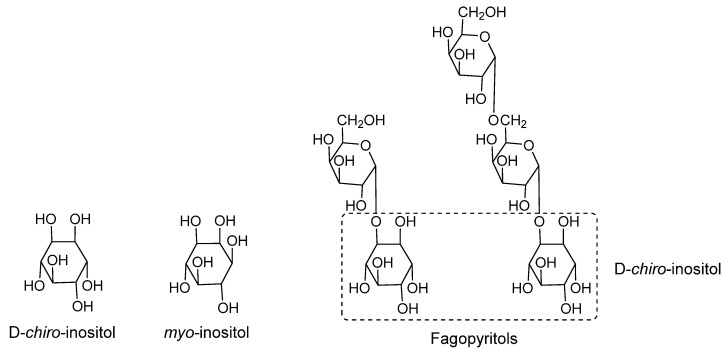
Identified molecules by gas chromatography in *C. ficifolia* extracts.

In 2006, Xia and Wang [41], in search of the identification of bioactive molecules, analyzed the profile of inositols and soluble carbohydrates of the hydro-methanolic extract (70%) of the fruit of *C. ficifolia* through its derivatization by silylation and identification and quantification by gas chromatography. The identified carbohydrates were D-*chiro*-inositol (DCI) (2.9 ± 0.2 mg/g), *myo*-inositol (7.8 ± mg/g), fagopyritols (33.6 ± 3.9 mg/g) and sucrose (126.8 ± 9.1 mg/g) [42] (Figure 1).

Previous research describes inositol molecules, especially DCI, as mediators of insulin action through the administration of synthetic D-*chiro*-inositol (at doses of 10 and 15 mg/kg) in Streptozotocin diabetic rats, where a decrease of between 30 and 40% in the blood glucose concentration was observed [43,44]. Xia and Wang employed the extract of *C. ficifolia* containing 10–20 mg/kg DCI and administered it orally to Streptozotocin-induced diabetic rats. The effect of the *C. ficifolia* extract was favorable by significantly reducing blood glucose and increasing the levels of liver glycogen, total haemoglobin, and plasma insulin. The effect of the administration of *C. ficifolia* is comparable to that of synthetic DCI, therefore, the *C. ficifolia* extract can be considered a natural source of DCI with hypoglycemic activity. These findings suggest that the fagopyritols also found in the *C. ficifolia* extract could have a certain hypoglycemic effect by being hydrolyzed in the in vivo model and releasing DCI units [41]. Fagopyritols have also been identified in tartary buckwheat (*Fagopyrum tataricum*) and are described as having antidiabetic activity [45]; thus, they also may contribute to the bioactivity of the *C. ficifolia* extract.

Fortis-Barrera et al. [46] investigated the antioxidant and anti-inflammatory effect of the aqueous extract of *C. ficifolia* and of synthetic DCI on adipocytes. Both, the extract and the synthetic DCI reduced oxidative stress by decreasing H_2_O_2_ levels, increasing glutathione peroxidase enzyme activity, and changing the GSH/GSSG ratio. DCI caused the decrease in mRNA expression and the secretion of tumor necrosis factor-alpha (TNF-α), interleukin 6 (IL-6), and resistin. On the other hand, the *C. ficifolia* extract gave rise to a diminution in resistin levels and an increment in IL-6 levels. Therefore, only DCI possesses an insulin-mimetic action, and the anti-inflammatory and antioxidant effect of the *C. ficifolia* extract can be largely explained by its DCI content. Thus, DCI may have a beneficial effect in the treatment of obesity and T2DM.

In another investigation, the anti-inflammatory and antioxidant activities of a precipitate from a fraction of the aqueous extract of the fruit of *C. ficifolia* with a content of 3.31 mg/g of DCI [47] were also determined in healthy and Streptozotocin-diabetic mice. The results showed similar behavior to that found by Fortis-Barrera et al. [46], where the administration of the fraction produced an increase in GSH levels and a decrease in malondialdehyde (MDA) in the liver. TNF-α levels were diminished and IL-6 and interferon-gamma (IFN-γ) levels were increased in serum. This increase observed in the mediators may be due to the inflammatory effect of Streptozotocin or as a predictor of toxicity of the extract in mice. Hence, in addition to its hypoglycemic activity, the authors describe the extract of *C. ficifolia* as having antioxidant and anti-inflammatory properties, suitable for the control of DM.

**Figure 2:**
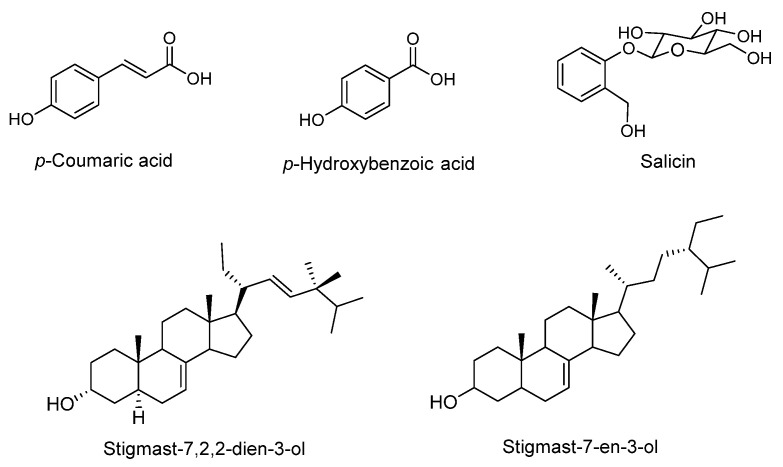
Main molecules identified in the aqueous extract of the fruit of *C. ficifolia*.

A later study identified the following five of the main components of the aqueous extract of *C. ficifolia*: *p*-coumaric acid, *p*-hydroxybenzoic acid, salicin, stigmast-7,22-dien-3-ol, and stigmast-7-en-3-ol [48] (Figure 2), while DCI was not detected in the extract. The extract of *C. ficifolia* was administered to Alloxan-induced diabetic mice and a histological analysis of the mouse liver revealed the accumulation of glycogen. Additionally, the extract of *C. ficifolia* exerted a protective effect on hepatocytes due to the absence of neither necrosis nor cell alterations, that were observed in the liver tissue of the mice. In addition, an increased glycogen synthase and decreased glycogen phosphorylase were recorded; therefore, the hypoglycemic effect of this extract may be partially explained by the glycogen accumulation in the liver. Compounds such as *p*-coumaric acid, *p*-hydroxybenzoic acid, and salicin have antioxidant, anti-inflammatory and gluconeogenesis inhibitory effects reported previously, contributing to the hypoglycemic effect of the *C. ficifolia* extract [48].

Therefore, since the antidiabetic activity is related to the antioxidant and anti-inflammatory process, Table 1 presents the bioactive molecules identified in the extracts of *C. ficifolia* with multiple actions that could be useful in the treatment of DM.

### 3.2. Cucurbita Maxima Duchesne

*Cucurbita maxima* is native to the Americas and was cultivated by the ancient civilizations of Central and South America more than 7000 years ago. Other varieties can be found in Australia, Africa (Zambia, Nigeria, and Zimbabwe), Asia (China, India, Iran, Afghanistan), and Europe (Spain and Turkey) [49,50]. It is known as winter *squash*, *butternut squash*, *kalabazeam maboga*, *zapallo*, *parangi*, *giromon*, *cocozza* [51], *giant pumpkin*, *hubbard squash*, *kabocha squash* or *pumpkin* [30]. Traditionally, the leaves, fruits and seeds are used to treat various ailments. The seeds are consumed orally for the treatment of intestinal worms, parasites [52,53], constipation [52], kidney problems [54], and prostatitis [55]. The leaves have been utilized for the treatment of anemia [56] and head and lung cancer [57]. Oral consumption of the fruit in traditional medicine for the treatment of urinary disorders [54], blood pressure control, and constipation has been recorded [58]. In Mexico, China, and Iran the juice of the fruit of *C. maxima* is employed for control of the blood glucose level [30,59].

Several investigations have been conducted to characterize the bioactivity of the flowers, seeds, and fruit of *C. maxima*. Among these studies, its antimicrobial, antioxidant [60] anti-inflammatory [61,62], cytotoxic [63] and antidiabetic [64] activity stands out.

In vitro, in vivo, and patient trials have been developed to determine the antidiabetic effect of extracts from leaves, flowers, and seeds of *C. maxima*. Some approaches for the treatment of T2DM focus on the decrease of the postprandial hyperglycemia effect. Inhibition of the enzymes responsible for hydrolyzing carbohydrates, such as α-glucosidase and α-amylase, contributes to the control of postprandial hyperglycemia [65]. Al-Shaheen et al. [64] evaluated the antioxidant and the inhibitory capacity in the α-amylase enzyme of the methanolic extract of *C. maxima* leaves. The extract was shown to have detectable and higher antioxidant activity than the positive controls (BHT, BHA and vitamin C), with an IC_50_ value of 125 µg/mL. Similarly, the extract exhibited the potential to inhibit the enzyme α-amylase by 97.01% at a concentration of 100 mg/mL and an IC_50_ value of 2.1 mg/mL.

In another research, in vitro tests also demonstrated the inhibitory activity of the aqueous extract of the seeds of *C. maxima* in the enzymes α-glucosidase and α-amylase, with IC_50_ values of 8.11 ± 0.36 mg/mL and 7.00 ± 0.29 mg/mL, respectively. Additionally, phytoelements such as Mg, Ca, Na and, K, which have been shown to inhibit the enzymes α-glucosidase and α-amylase, were detected in the aqueous extract of the seeds, where they probably contributed to the hypoglycemic effect of the extract [66].

**Figure 3:**
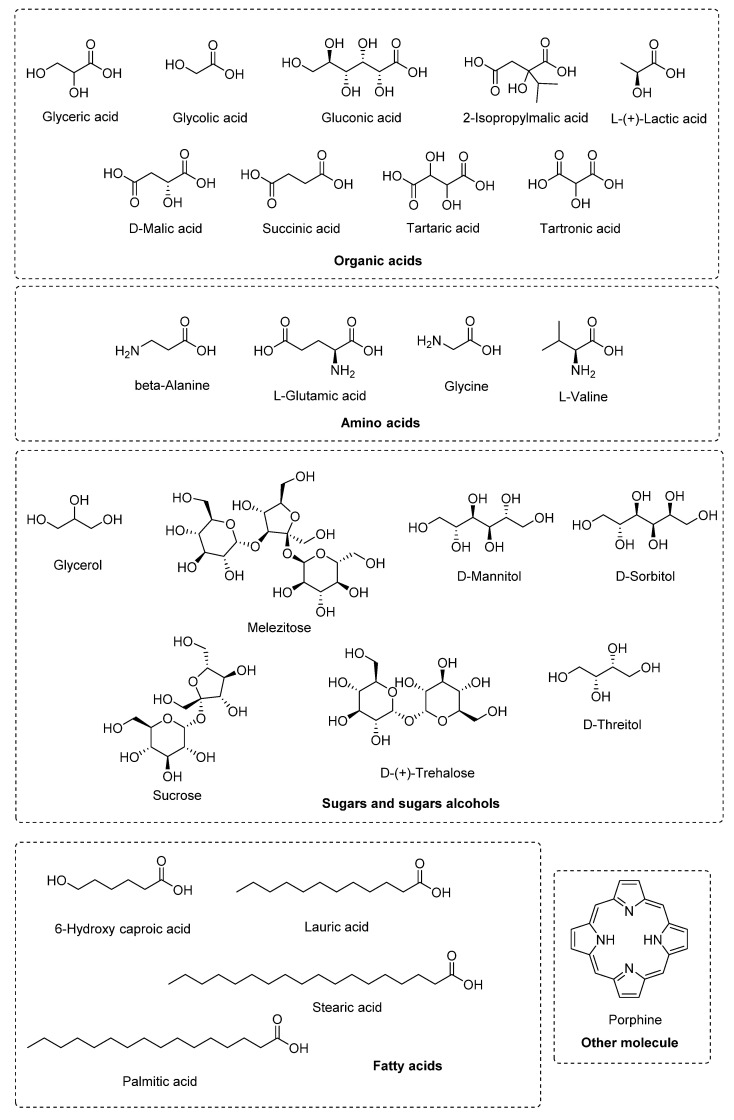
Conspicuous chemicals of the methanolic extract of the flowers of *C. maxima*.

The methanolic extract of the flowers of *C. maxima* possess an inhibitory effect on the enzyme α-glucosidase with an IC_50_ value of 610.52 ± 60.33 μg/mL. In this same study, the neuroprotective, hepatoprotective, and antihypertensive effect of the methanolic extract of the flowers of *C. maxima* was evaluated by inhibiting the enzymes acetylcholinesterase (AChE, IC_50_ = 460.78 ± 6.01 μg/mL), β-glucuronidase (64.69% ± 1.29 inhibition at 500 μg/mL), and the angiotensin converting enzyme (ACE, 46.63% ± 1.15 inhibition at 15 μg/mL). Thus, the methanolic extract of the flowers of *C. maxima* demonstrated antidiabetic, neuroprotective, and antihypertensive potential. Some of the most conspicuous phytochemicals of the extract were identified by gas chromatography, and included, nine organic acids, seven sugars and sugar alcohols, four amino acids, four fatty acids, and porphine [67] (Figure 3).

In vivo tests have been carried out with extracts from different parts of *C. maxima*, which have shown a hypoglycemic effect. Lal et al. evaluated the hypoglycemic effect of the fruit juice and the hydroethanolic extract of the aerial parts in Streptozotocin induced diabetic rats at a dose of 100 and 200 mg/kg for 28 days. The extract showed a higher hypoglycemic effect than the juice [68]. Similarly, in another study, the methanolic extract of the aerial parts of *C. maxima* was shown to have a hypoglycemic effect at 200 and 400 mg/kg administered to Streptozotocin induced diabetic rats for 14 days. The extract also revealed an antioxidant effect by increasing the enzyme GHS and catalase (CAT) and generated a protective effect by decreasing the levels of the enzymes glutamic oxaloacetic transaminase (SGOT), serum glutamine pyruvic transaminase (SGPT), and alkaline phosphatase (ALP), which were elevated in the control group of rats with diabetes. The antioxidant and antidiabetic effect of the extract was explained by the presence of polyphenols or polysaccharides in the extract [69].

**Figure 4:**
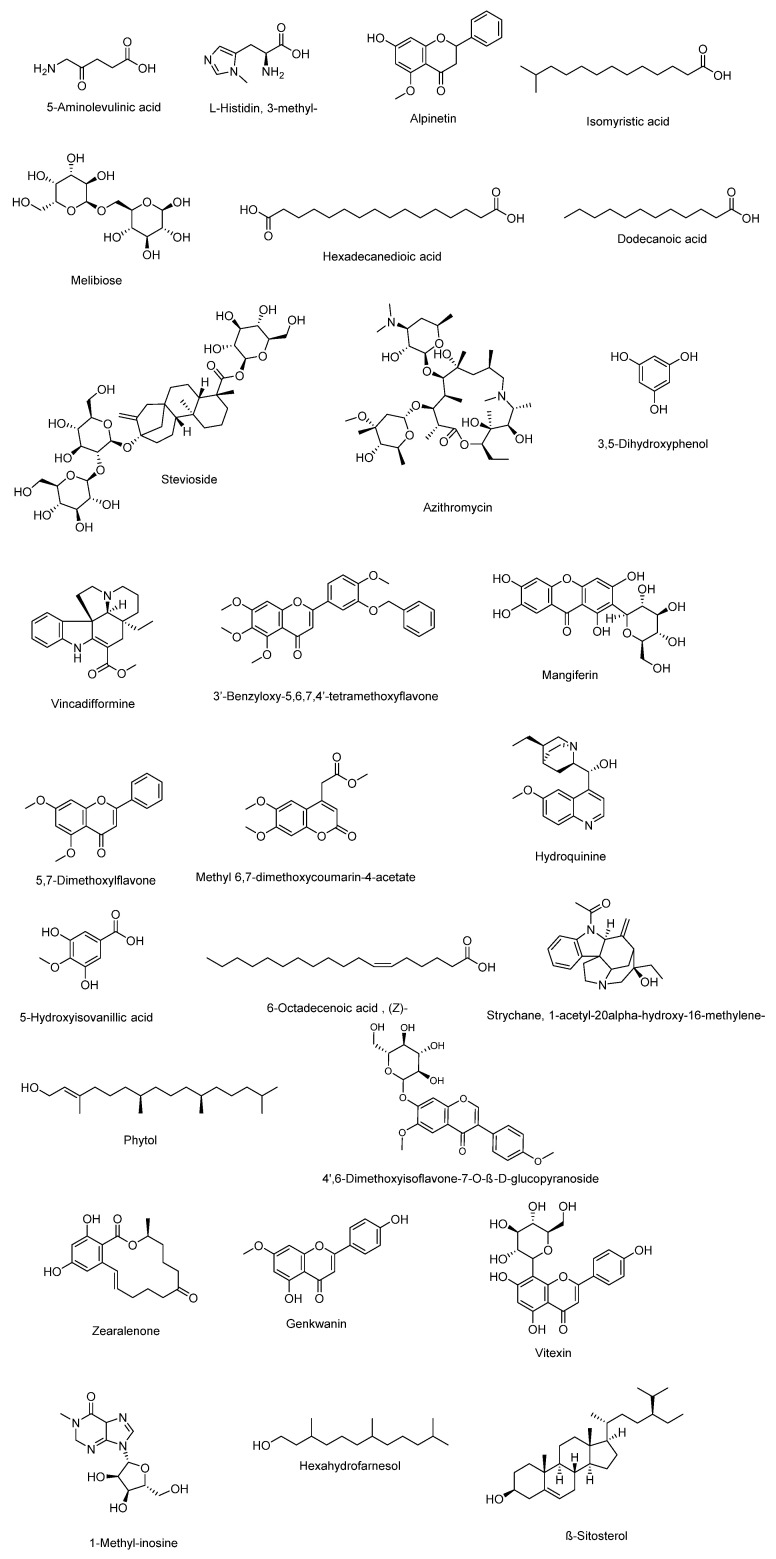
Molecules identified of the methanolic extract of the fruit of *C. maxima*.

Hepatoprotective potential was exhibited for the methanolic extract of the seeds [70] and the fruit [71] of *C. maxima*. The extract of the seeds was shown to prevent liver damage caused by diabetes, where a decrease in the levels of AST (aspartate aminotransferase), ALT (alanine aminotransferase), and ALP (alkaline phosphatase) was found. The methanolic extract of the seeds was also shown to have a renal protective effect by the decrease of creatinine (CRTN) levels and the increase of hemoglobin (Hb) and total protein (TPR) levels in diabetic rats. Concerning the methanolic extract of the fruit of *C. maxima*, it was also shown to have a protective effect on the pancreas against damage caused by diabetes. This hepatoprotective effect was determined by histopathological examination of the pancreatic islet cells from diabetic rats, in which, the disappearance of severe degenerative and necrotic changes in pancreatic cells was observed. Analysis by gas chromatography coupled to mass spectrometry revealed the presence of flavonoids such as vitexin and other flavone C-glycosides, carotenoids, and polyphenols, which may be involved in the stabilizing effect of the hepatocyte membrane and antioxidant activity that decrease the oxidative stress generated by diabetes. Other identified compounds include fatty acids, amino acids, alkaloids, disaccharides, terpenes, phytosterols, and saponins (Figure 4), which probably present a synergistic effect to protect against the liver and pancreatic damage caused by diabetes [71].

On the other hand, the hypoglycemic and antihyperlipidemic effect of petroleum ether, ethyl acetate and ethanolic extracts from the seeds of *C. maxima* in Streptozotocin induced diabetic rats was investigated [72]. All extracts, especially the ethanolic extract, exhibited a hypoglycemic and antihyperlipidemic effect. The oral administration of 200 mg/kg of extracts for 21 days potentiated an increase in insulin and HDL-cholesterol levels, while total cholesterol (TC), LDL, VLDV, and triglycerid (TG) levels decreased in the blood serum. In these in vivo tests, no death of any animal was reported at the administered concentrations, indicating the non-toxicity effect of the *C. maxima* extracts.

The hypoglycemic effect of the lyophilized fruit juice of *C. maxima* was evaluated in a clinical trial. A group of 20 patients, in which 12 were patients with T1DM, and eight patients with T2DM, were orally administrated with 5g of the lyophilized fruit juice every 12 h for 3 days. The lyophilized fruit juice of *C. maxima* showed a significant reduction in the blood glucose level and was rapid and effective, without presenting adverse effects [59].

### 3.3. Cucurbita Moschata Duchesne

Archaeological evidence indicates that the possible territories where *C. moschata* was domesticated are localized in Mexico (about 5000 B.C.), Peru (3000 B.C.) or Guatemala (2000 B.C.) [50]. Currently, *C. moschata* is widely distributed in Asia, America, and Africa [73]. Common names for *C. moschata* include *butternut squash*, *pumpkin*, *cheese pumpkin*, *golden cushaw*, *Japanese pumpkin*, *melon squash*, *musky gourd*, *musky pumpkin*, *musky squash*, *musky winter squash*, *seminole pumpkin*, *tropical pumpkin*, *winter crookneck squash*, *winter marrow*, *winter pumpkin*, *winter squash*, and *winter straightneck squash* [74]. *C. moschata* is used to prepare a variety of traditional dishes, but is also utilized in traditional medicine. The seeds are employed to relieve kidney affections, as an antiparasitic, and in the treatment of intestinal infections. The flowers are used topically to soothe minor wounds [74,75]. In China, the seeds of *C. moschata* are officially prescribed by traditional medicine for the treatment of DM [76]. Research on the bioactivity of *C. moschata* reveals that polysaccharides exhibit antioxidant [60], antibacterial [77], anti-obesity [78], antitumoral [79], antiparasitic [80], and hypoglycemic effects [81,82]. A number of studies have been carried out to delve into the antidiabetic properties of the fruits, flowers and stems of *C. moschata*. Several reports showed that hypoglycemic activity has been related to the carbohydrate content (Table 2).

Song and collaborators [83] identified, by gas chromatography, two galactose monosaccharides and glucose as components of the active aqueous extract of the fruit of *C. moschata*, with a presence of 86.4 and 13.6%, respectively. The aqueous extract was able to inhibit (97.4%) in a non-competitive mechanism, the enzyme α-glucosidase in a concentration of 0.7–0.9 mg/mL.

In addition to the identification of simple carbohydrates, the isolation and identification of two glyceroglycolipid tetrasaccharides from the fruit of *C. moschata* has been reported. The glyceroglycolipid tetrasaccharides QGMG2 and QGMG3 (50 mg/kg) were able to decrease serum glucose levels in a similar way to Metformin, when administered in diabetic mice induced by Streptozotocin and with a high-fat diet. The compound QGMG-3, with a side chain of three unsaturations, exhibited higher activity than QGMG-2, with a side chain of two unsaturations [81]. Similarly, Jin et al. evaluated the ethanolic extract of *C. moschata* in mice with Alloxan-induced diabetes. The fraction composed of glucose, galactose, arabinose, and rhamnose produced a significant reduction in blood glucose levels, from 15.90 ± 3.21 mM to 7.19 ± 2.54 mM [84].

Other evidence of the hypoglycemic effect of the *C. moschata* extract was shown by Zhang et al. [85]. These authors investigated the effect of the administration of a fraction of water-soluble carbohydrates obtained from the hydroethanolic extract (80%) of the *C. moschata* fruit in Alloxan-induced diabetic rabbits in a dose of 75 mg/kg for 21 days. The results indicated that this polysaccharide fraction improves the body-weight loss of rabbits and also a reduction in the levels of blood glucose (BG), total cholesterol (TC), triglycerides, and glycated hemoglobin (HbA1c) were observed. Additionally, in the analysis of the pancreatic tissue, the water-soluble carbohydrate fraction exerted the regeneration of damaged pancreatic islets by stimulating β-cell proliferation. The chemical analysis of this polysaccharide fraction revealed the presence of glucose, galactose, arabinose, rhamnose, and a small amount of hexuronic acid.

**Figure 5:**
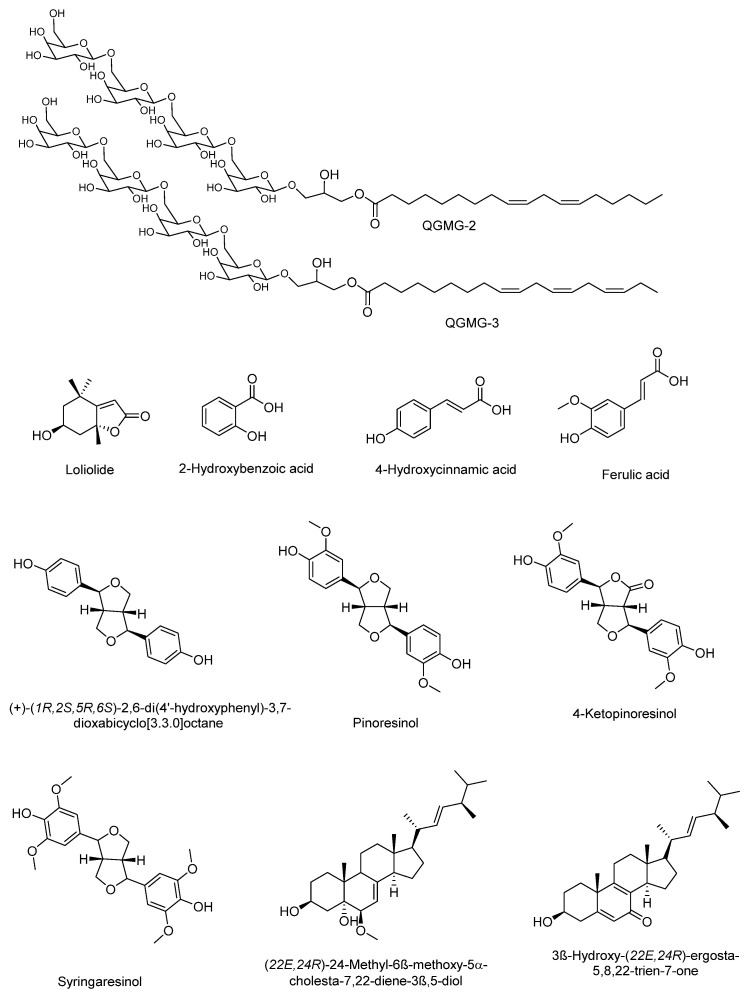
Molecules isolated of the extract of stem of *C. moschata* with hypoglycemic activity.

Chang et al. [86] reported some components of the stem of *C. moschata* and the hypoglycemic effect and the mechanism of action. Ten compounds from two fractions of the methanolic extract of the stem of *C. moschata* that presented hypoglycemic activity in Streptozotocin-induced diabetic mice were identified. These compounds include apocarotenoids (loliolide); phenolics [2-hydroxybenzoic acid; 4-hydroxycinnamic acid; ferulic acid]; lignans [(+)-(1*R*,2*S*,5*R*,6*S*)-2,6-di(4′-Hydroxyphenyl)-3,7 dioxabicyclo [3.3.0]octane; pinoresinol; 4-ketopinoresinol; syringaresinol]; and steroids [(22*E*,24*R*)-24-Methyl-6*β*-methoxy-5*α*-cholesta-7,22-diene-3*β*,5-diol; 3*β*-Hydroxy-(22*E*,24*R*)-ergosta-5,8,22-trien-7-one] and are shown in Figure 5. Steroid compounds 24-methyl-6*β*-methoxy-5*α*-cholesta-7,22-diene-3*β*,5-diol, and 3*β*-hydroxy-(22*E*,24*R*)-ergosta-5,8,22-trien-7-one promoted glucose uptake in normal hepatocytes in a similar way to that of insulin. The mechanism of action of these compounds may be mediated by AMPK activation. On the other hand, despite the structural difference of compounds ferulic acid, syringaresinol, and 24-methyl-6*β*-methoxy-5*α*-cholesta-7,22-diene-3*β*,5-diol, they showed an insulin-sensitization and/or insulin-substitution function in insulin-resistant cells. Thus, the stem of *C. moschata* contains compounds with the potential to control T1 or T2DM [86]. The hypoglycemic effect of the fruit of *C. moschata* has also been demonstrated for the seeds. Marbun et al. [87] reported the antidiabetic activity of the ethanolic extract of the seeds and pulp of *C. moschata*, which was comparable to the effect of Metformin in Streptozotocin-induced diabetic mice. Preliminary tests revealed the presence of flavonoids, terpenes, saponins, and tannins in both extracts.

### 3.4. Cucurbita pepo L.

*C. pepo* is a Cucurbitaceae known as *pumpkin* that has been part of the human diet for more than 10,000 years and that possesses the oldest record of domesticated Mesoamerican species, over other members of the Cucurbitaceae family such as *C. moschata*, *C. argyrosperma*, and *Lagenaria siceraria*. Recent data suggested that pre-Columbian Mexican civilizations probably cultivated *cucurbits* prior to corn [88]. *C. pepo* is native to Mexico and today is cultivated in association with other plants as part of the cultivated corn field (milpa) or as a single crop [89]. The flowers and fruits are consumed in Mexico through the preparation of candies, cakes, or traditional dishes [90]. In traditional medicine, *C. pepo* had been used largely in a number of countries around the world as a diuretic and antihelminthic. However, one of the most common uses is in the treatment of irritable bladder for enlarged prostate-gland and micturition problems. Nonetheless, even these treatments with *C. pepo* seeds diminish micturition, it has not very effective in reducing the expanded size of the prostate gland [91].

**Figure 6:**
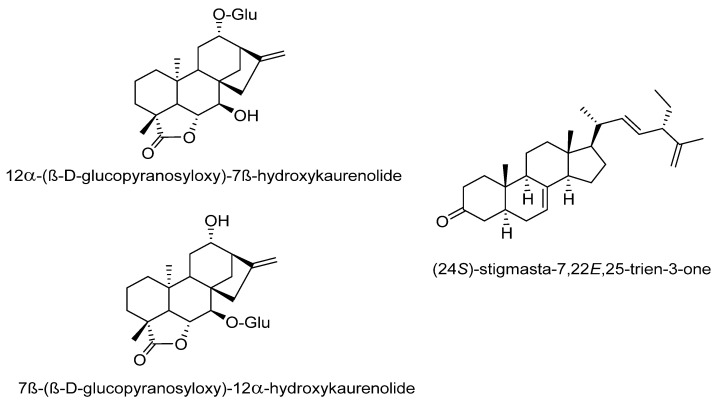
Novel isolated compounds of *C. pepo* seeds.

Regarding the phytochemicals reported in *C. pepo*, the fruit is characterized by a high total carotenoid content of 171.9 to 461.9 μg·g^−1^, but also by a low content of fat (2.3%), and a poor polyphenol content, which was calculated as 0.02 mg GAE/100 mg sample by fresh fruit [92]. Nevertheless, 57 phenolics and other polar compounds were identified by HPLC coupled with two different detection systems: diode array (DAD) and quadrupole time-of-flight (Q-TOF) mass spectrometry. These phenolics were grouped as hydroxycinnamic acids and derivatives, hydroxybenzoic acids and derivatives, flavones and glycosides, flavonols and glycosides, organic acids and derivatives, amino-acids and derivatives, and nucleosides [93]. Studies on the chemicals present in the seeds of *C. pepo* are numerous and varied. Some variations in the composition of the main chemicals in the seeds have been detected. However, the seeds of *pumpkin* content are approximately 50% oil and are a good source of phytosterols, carotenoids, fatty acids, and vitamins. The seeds of *C. pepo* have been characterized by their tocopherol content, where γ-tocopherol is more abundant than α-tocopherol. The oils of seeds contain phenolics such as tyrosol, vanillic acid, vanillin, luteolin, and sinapic acid, where tyrosol was the most abundant compound detected in an amount of 1.6 mg/kg to 17.7 mg/kg [94]. Other reports found that *p*-hydroxybenzoic acid was the most conspicuous phenolic acid in all parts of the seeds of *C. pepo*, such as kernels and the hulls [51]. The main fatty acids reported in the *pumpkin* seeds are oleic, linoleic, linolenic, palmitic, and stearic acids, while among the most conspicuous carotenoids, we find lutein, α-carotene, β-carotene, auroxanthin, flavoxanthin, luteoxanthin, β-carotene violxanthin [92]. The terpenes found in the *C. pepo* seeds were studied by Kikuchi et al. [95], who reported the presence of 3-*p*-aminobenzoyl multiflorane-type triterpenes, 7-epi zucchini factor A and debenzoyl zucchini factor B. Additionally, five malutiflorane-type triterpenoids, two new *ent*-kaurane diterpene glycosides; and a new steroid, (24*S*)-stigmasta-7,22*E*,25-trien-3-one, were identified (Figure 6). From the sprouts of *C. pepo*, two novel multiflorane *p*-aminobenzoates were isolated: 7-epi zucchini factor A (**1b**) and debenzoyl zucchini factor B. None of these compounds were found in adult plants [96]. However, the triterpene squalene is one of the most conspicuous in *pumpkin* seeds and has been employed as a marker. Squalene has also been recognized to possess anticancer, antibacterial, and antifungal properties and plays an important role in the metabolism of cholesterol and steroid hormones [97]. The sterols are another group of compounds that are considered as prominent in *C*. *pepo*, particularly Δ7 and Δ5 sterols. Δ stigmastatrienol and Δ spinasterol have been considered as the main sterols isolated in seeds of *C. pepo*, followed by Δ stigmasterol, stigmastadienol, and β-sitosterol [98].

On the other hand, some pharmacological properties of *pumpkin* have been reported, such as antimicrobial, hepatoprotective, lipid lowering, antioxidant, antihypertension, anti-inflammatory, and antidiabetic properties [99]. However, these latter antidiabetic properties have been acquiring relevance in recent years, because of the traditional use of *C. pepo* in the treatment of DM in Mexico, in which local healers recommend the ingestion of crude aqueous extracts of *pumpkin*, which represents a source which is available for the entire population; therefore, it comprises an alternative treatment for the growing population that suffers from DM, especially in Mexico.

The hypoglycemic activity (blood sugar lowering) of *C. pepo* extracts was demonstrated in Alloxan-induced diabetic rats and rabbits, and interestingly, these active hypoglycemic properties of pumpkin were identified in the pulp and seed extracts, in contrast with those of the leaves and stems [99]. Pumpkin powder was administrated during 4 weeks to Alloxan-induced diabetic rats, where the results showed a significant reduction in the levels of glucose, cholesterol, triglycerides, low density lipoprotein (LDL) and C-reactive protein (CRP) levels. However, even the pumpkin powder increased the levels of blood insulin and high density lipoprotein (HDL), though not significantly. The histological analysis evidenced the restoration of the pancreatic tissue caused by the administration of the pumpkin powder, due to the increase in the diameter and number of the Langerhans islets observed in rats fed with pumpkin [100].

Other studies revealed that the antidiabetic properties of pumpkin have been observed through the antioxidant activities of the raw fruit of the pumpkin. Especially, the content of tocopherols was attractive due to its already known high antioxidant properties. Therefore, Bharti et al. [101] determined the content of tocopherol in the seeds of pumpkin by HPLC (107.4 ± 2.9 mg/100 g) and tested this on diabetic rats. In addition to glycemic profile, a variety of criteria to establish the possible impact of tocopherols on the physiology of DM was included in those investigations, such as insulinemic and lipidic profiles; histological studies; molecular docking investigations; endogenous enzymatic and nonenzymatic antioxidant levels, and GLP-1 content in the cecum. The results indicated that tocopherol induced a significant glucose lowering together with a reduction of insulinemia and a decrease in insulin resistance at the higher dose employed (5 g/kg body weight). However, the tocopherol did not exhibit benefits in the lipidic profiles. Concerning the histological studies, tocopherol induced a significant improvement in the number of pancreatic islets as well as in the grade of insulitis. In the docking studies, the tocopherol isomers exhibited considerable interaction in the active sites of the proteins. A notable reduction was also observed in the oxidative markers such as SOD, catalase, GST, TBARS, total thiols, and GSH. Finally, a remarkable increase of GLP-1 content in the cecum by the administration of tocopherol was recorded in diabetic rats along with cecal-tissue enlargement.

On the other hand, polysaccharides have been identified as crucial for the hypoglycemic properties of pumpkin. In the case of protein-bound polysaccharide (PBPP) isolated from the aqueous extract of pumpkin fruit, this showed a dose-dependent hypoglycemic effect, because of the marked effect exhibited by the dose of 1000 mg/kg when administrated in diabetic rats, over the 500 mg/kg dose, and also because of the comparison with the control group (Glibenclamide). The PBPP from pumpkin was able to increase the levels of insulin in serum and also stimulate pancreatic cells. With these characteristics, PBPP may be able to be considered as a future novel antidiabetic agent [102]. Fractions with a content of polysaccharides from pumpkin powder were able to significantly reduce the blood glucose levels in diabetic mice, but were not able to stimulate cells in the islets of Langerhans. However, pumpkin polysaccharides may play an important role in the recovery of liver function and glucose utilization. The most active fraction of polysaccharides was made up of four monosaccharides, including glucose, galactose, arabinose, and rhamnose in a proportion of 2.0:1.0:1.5:2.5 [84].

Another work reported the extraction of a polysaccharide denominated PP-PE with a molecular weight of 2.4 × 10^4^, and composed of α-(1→6)-glucan branched at the C3 position and α-(1→4)-galactan, obtained by the hot water extraction of *C. pepo*. This PP-PE was tested for in vivo antidiabetic activities in Alloxan-diabetic mice (a dose of 100 mg/kg/day), and in in vitro assays for measuring α-glucosidase inhibitory activity and α-amylase inhibitory activity. The results revealed that the blood glucose level decreased after 7 days of PP-PE treatment and a normal histological structure of β-cells at the central zone in the islet of Langerhans was also observed. In the in vitro tests, the PP-PE was able to inhibit α-glucosidase and α-amylase activities, and the IC_50_ values were calculated as 110.32 ± 7.08 and 103.06 ± 1.60 mg/mL, respectively. Hence, C. *pepo* could be considered as promising in the treatments of diabetes [103].

The hypoglycemic properties of pumpkin have been reported mostly in experimental animals, but also some reports indicated the possible hypoglycemic effect of pumpkin in humans, in patients with T1DM and T2DM [104]. In addition to this, the consumption of pumpkin as a dietary component and as a source of fiber has been analyzed not only in the treatment of DM, but also to prevent the risk of other diseases such as obesity, atherosclerosis, heart diseases, colon cancer, and colorectal cancer. From the nutritional and industrial perspective, pumpkins are attractive due to the fact that they are a good source of carotenoids, pectin, mineral salts, and vitamins. Recent studies propose that the raw materials of pumpkin can be useful to produce pumpkin flour for enriching food with by a high content of dietary fiber. In this respect, pumpkin emerges as a *cucurbit* that is useful not only from the pharmacological perspective for the treatment of DM, but also as a central dietary component in diabetic population [105].

### 3.5. Ibervillea sonorae (S. Watson) Greene

*Ibervillea sonorae*, known as *wareke* o *wereke*, is a perennial plant distributed in the northern states of Mexico, including Sinaloa, Sonora, and Baja California, and the southern regions of the U.S. Some indigenous tribes of that Mexican region, such as the Mayo, Opata, Seri, and Yaqui, have traditionally used *wareke* for the treatment of skin diseases. Nevertheless, since pre-Hispanic times, knowledge about its empirical use for minor skin infections, arthritis, rheumatism, and heart conditions have been recorded [106].

**Figure 7:**
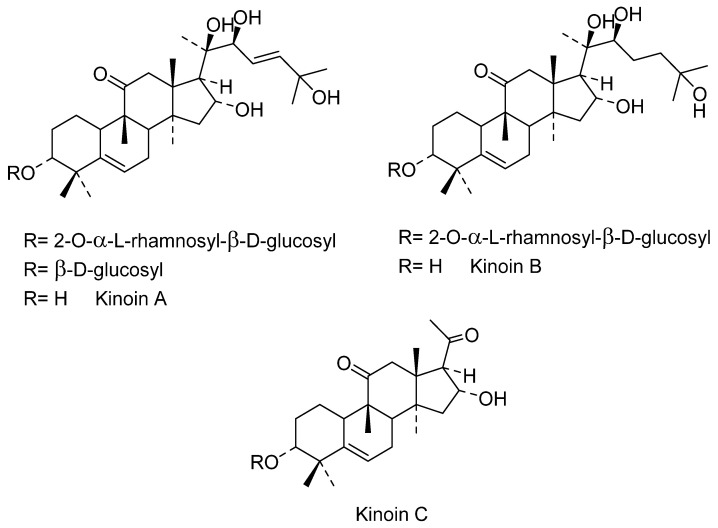
Molecules of the type of the cucurbitan series isolated from *I. sonorae*.

In the 20th century, different activities have been found in the extracts of the roots, probably because these organs are the most notorious parts of *I. sonorae*, exhibiting a tuberculous appearance (root tuber) that is usually marketed. In 1993, Domínguez et al. [107,108] described the first formulas of the main chemical components of these tuberous roots of *I. sonorae*, describing the isolation and characterization of six compounds of the type of the cucurbitan series of tetracyclic system of 3-glycosides-tetramethyl-19-norpregnenedione-17-2,3,6,-trihydroxy-6-methyl cucurbitacin Kinoin A and Cucurbitacin B; Kinoin A and B glycosides; hexanorcucurbitacin Kinoin C [3a,16adihydroxy-4,4,9,14-tetramethyl-(9~,10~)-19-norp~gn-5-ene-l 1,20-dione] and Kinoin C triterpenes (Figure 7). These cucurbitacins were named in honor of Father Quino, an Austrian-Italian Jesuit missionary, explorer, cartographer, geographer, and astronomer, distinguished among the indigenous people in the present area of northwestern Mexico and in the southwestern U.S. for his methods of evangelization, the founder of 20 missions, and known for his ability to establish relationships between indigenous people and the religious institutions that he represented [107].

In recent decades, a number of reports have pointed out that the *wereke* were extensively utilized in the traditional medicine of the State of Sonora, Mexico, in a variety of uses such as a topical antibiotic, cathartic, antirheumatic, antidiabetic, and as anticancer. Particularly, this latter activity motivated Torres-Moreno et al. [109] to isolate the secondary metabolites cucurbitan-type triterpenes, Kinoin A and 3β−(2-O-α-L-rhamnosyl-β-D-glucosyl) (Kinoin B), to establish the antiproliferative and induction of cell death by apoptotic activities.

Other studies found that *wereke* has anticancer potential, in that it possesses a cytotoxic effect on T47D cancer cell lines [110] due the presence of cucurbitan-type triterpene glycosides. The isolation of Cucurbitacin IIb and the effect of the reduction in the growth of cancer cells, as well as the induction of apoptosis in HeLA and A549 at low doses, were reported by Robles-Zepeda et al. [111]. The structural differences of Cucurbitacin IIb with the less active Kinoin A and Kinoin B diglycoside, were the subject of study, where the presence in the molecule at position 2 of a hydroxyl group with β orientation, was found to enhance antiproliferative activity [112]. Recently, Zepeda et al. [113] were able to use the roots of *I. sonorae* in the therapy of some cancers. These authors prepared and tested two phytopreparations, Phyto-ison-EtOH and Phyto-ison-AcOEt, measuring their antiproliferative properties. From Phyto-ison-EtOH a new cucurbitacin (25-anhydro-Kinoin A diglycoside) was isolated and characterized, together with active CIIb and the diglycoside of Kinoin B. By means of experiments with the APCI-IT MsN of the two phytopreparations, the presence of Kinoin A, 3-glucoside Kinoin B and 3-diglycoside Kinoin A was also detected.

**Figure 8:**
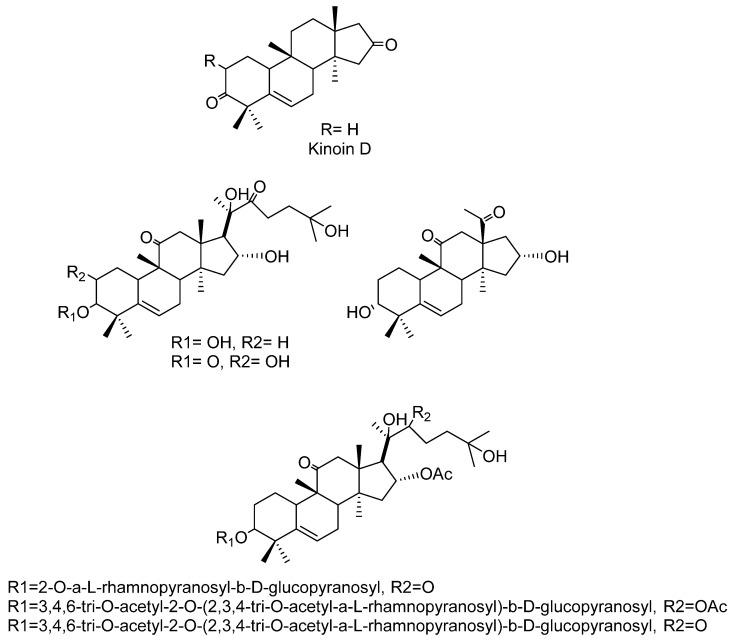
Kinoin D octanocucurbitacin-type triterpene with anti-inflammatory activity and other compounds isolated of *I. sonorae*.

On the other hand, antibiotic, antifungal, anti-inflammatory, and antiviral properties are also attributed to *wereke* [30,114]. Martínez Vázquez et al. [115] isolated and characterized a new octanocucurbitacin-type triterpene (Kinoin D) with anti-inflammatory activity from the extract with ethyl acetate and the other compounds already isolated from *I. sonorae* (Figure 8). Sinagawa-Garcia et al. [116] carried out a bromatological analysis that indicated the nutritional composition of *wereke* roots. In which the presence of an active proteinase at alkaline pH and the inhibitory activity of trypsin were reported, to our knowledge for the first time, in *wereke*.

**Figure 9:**
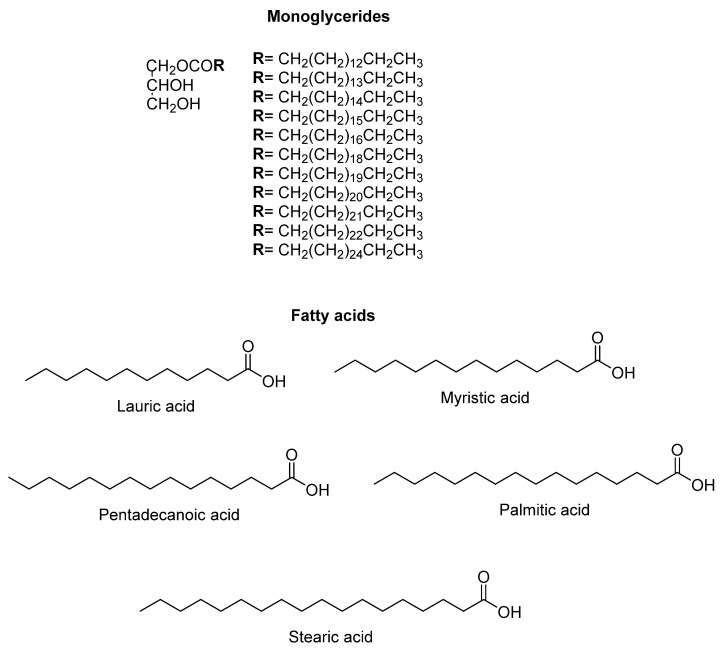
Monoglycerides and fatty acids with hypoglycemic effects of *I. sonorae*.

Nonetheless, in the 20th century, the decoction of *wereke* roots became one of the most used remedies in Mexico for the treatment of DM, not only in the northern region of Mexico, but also extending through the country. The series of works conducted by Alarcón-Aguilar et al. (2002–2007) [117,118,119] showed that the aqueous decoction of *I. sonorae* roots reduces the blood sugar concentration, validating the traditional use of the plant. Hence, according to the first of these works, the freeze-dried decoction (traditional preparation) of *I. sonorae* decreases the blood glucose levels in healthy mice and mild Alloxan-diabetic mice and rats. However, no changes in the levels of hyperglycemia were observed in severe Alloxan-diabetic rats [117]. In the second study, *wareque* root was administered, as in the traditional preparation, as the raw extract (juice), as the dichlormethane extract, and as a methanolic extract in a model of acute and chronic administration. At a dose of 600 mg/kg, the traditional preparation, the raw extract and the methanolic extract caused significant reductions of glycemia in healthy mice after intraperitoneal administration. However, the dichlormethane extract caused severe hypoglycemia that produced lethality in all of the animals treated with the same dose. Consequently, the dichlormethane extract was administered at a dose of 300 mg/kg to diabetic rats for 41 days. The levels of total cholesterol and uric acid did not exhibit any change, while the levels of glycemia, body weight, and triglycerides were incremented in comparison with those of the diabetic control group [118]. In the third work of the series, from the *wereke* roots, 11 compounds were isolated of the monoglycerides type, in addition to five fatty acids that showed hypoglycemic effects when administered in different mixtures to healthy and Alloxan-induced diabetic mice (Figure 9).

Hypoglycemic and anti-obesity effects were caused by the aqueous extract of *I. sonorae* incorporated into the diet of the mice. The *wereke* extract also promoted a decrease in food intake with a subsequent decrease in the body weight, and a decrease in obesity levels in mice treated with prepared diets [120]. In a different investigation, Zapata Bustos et al. [121] demonstrated that the aqueous extracts of the tuberous roots of *I. sonorae* induce the consumption of glucose in human adipocytes, which is the mechanism by which these roots have antidiabetic properties. In this work, the presence of flavonoids, phenolic compounds, and high amounts of gallic acid were detected. From another point of view, in recent years, the anti-hyperglycemic mechanisms of *I. sonorae* were studied by enzymatic inhibition studies that revealed that the aqueous extract of *I. sonorae* was able to inhibit the enzyme α-glucosidase in a concentration-dependent manner and competitive inhibition. Additionally, the *wereke* aqueous extract stimulates insulin secretion in vitro from RIN-m5F pancreatic *β* cells, which allows the proposal of the use of *I. sonorae* as a supplement in order to control the glucose blood levels of patients, as an alternative to combat the potential contraindications of *wereke* [122].

Estrada-Zúñiga et al. [123] established the culture of calluses from explants of *I. sonorae* leaves and determined the amounts of stearic, palmitic, lauric, myristic, pentadecanoic fatty acids, and the content of phenolic compounds. In terms of the complex chemical composition of these tubers, ketones, alkanes and amides, palmitic acid, and methyl palmitate, are noteworthy, which are associated with hypoglycemic activities [124]. In Table 3, the 33 compounds identified by GC-MS are found.

### 3.6. Sechium edule (Jacq.) Sw.

*Sechium edule*, popularly known as, *chayote*, is a native, perennial climbing plant domesticated by the Aztec and Maya civilizations. *Chayote* is an important element of the Mexican diet and is employed to prepare salads, jams, sweets, or desserts [125]. At present, *chayote* is cultivated in tropical and subtropical areas worldwide and is known by different names depending on the region where it is found, such as *cidrayote, chiote, cho-cho, choko, chow-chow, christophene, custard, hayatouri, huisquil, mango squash, mirliton, sayote, vegetable pear*, and *xuxu* [126]. *Chayote* is recognized in a wide range of shapes and sizes, but today it can be identified due to the existence of data on its genome and its proteome [127].

In recent years, *S. edule* has been investigated due to the therapeutic and nutritional potential of its natural product content [128]. Among some current uses of *chayote* are: its employment in the food industry due the production of starch; in the cosmetic industry, as an ingredient in moisturizers, cleansers, sun lotions, toothpastes, mouthwash, shaving creams, deodorants, and shampoos; and in the animal nutrition area due to its promotion of growth in pigs, because the *chayote* meals prepared with fruits and leaves were found to replace the standard grower ration in the diet of the pigs without any secondary effects; and this may be one of its most salient uses: in traditional medicine [129]. *Chayote* has been widely utilized in the treatment of diabetes, followed by uses such as a diuretic, in renal calculi, arteriosclerosis, hypertension, vermifuge, leprosy, and asthma [130,131].

A variety of in vitro and in vivo tests have been conducted in order to detect the pharmacological properties of *S. edule* in various organs or systems, such as the cardiovascular and the central nervous system, the gastrointestinal system, and in the liver and kidney, but very few of these are bioguided in order to identify the bioactive compounds. The phytochemical content of the *chayote* is diverse and the chemicals that have been identified are alkaloids, saponins, phenolic acids, flavonoids, carotenoids, coumarins, cucurbitane triterpenoids, and phytosterols [129]. According to some authors, the flavonoid and triterpene content that are the most relevant for biological activities in *S. edule*; thus, some of the main flavonoids identified in *S. edule* are apigenin 6-*C-β-D*-glucopyranosyl-8-*C-β-D*-apiofuranoside, diosmetin 7-*O*-rutinoside, luteolin 7-*O*-rutinoside, luteolin 7-*O-β-D*-glucoside, and apigenin 7-*O*-rutinoside [125]. In the same manner, the triterpenoids in *S. edule* are characterized by the presence of cucurbitacins such as the cucurbitancins B, E, P, and L [132].

On the other hand, as reported by Siahaan et al., flavonoids comprise the most relevant chemical content for the hypoglycemic effects observed in *S. edule*. A significant decrease in the blood sugar levels of mice was observed after the administration of the ethanolic extract of *S. edule* in a 200 mg/kg dose. However, no changes were observed in the activity of the glutathione peroxidase enzyme. Additionally, a difference in the diameter of the pancreatic β-cell was recorded in *chayote*-treated mice [133]. In a subsequent study, this same research group studied the anti-hypoglycemia and antioxidant activities of *S. edule* in a rat model of T2DM. The ethanol extract and the ethyl acetate fraction of *chayote* possess antioxidant and anti-insulin resistant activities as they were able to reduce the levels of blood sugar and increase the antioxidant level of superoxide dismutase (SOD) [134].

Because the fruit of *S. edule* is widely recommended in Mexico for reducing not only the glucose blood levels, but also the risks related to diabetes, a series of works were carried out during the last decades. Dire et al. reported that the *Sechium edule* extract was able to modify the biodistribution of certain metabolites in the pancreas, such as AGE (Advanced Glycation End Products), which are characterized by inducing general inflammation in addition to other anomalies related to DM. *Chayote* modified and inactivated these molecules in an in vivo model with Windstar rats [135]. In another work, the relation between DM and other diseases and risk factors usually present, such as obesity, fatty liver, and hypertension, originated the search for natural alternatives that could reduce one or some of these risk factors. Hence, *S. edule* sprouts, as well as its fresh leaves, were prepared to be macerated and tested in fatty liver, finding mixtures of polyphenols, which reduce the accumulation of lipids in the liver, and in an in vitro model of fatty acid accumulation induced in HepG2cells [136]. Additionally, because it has been revealed in recent years that diet is crucial to controlling diabetes and related illnesses, a number of food supplements have been proposed. *Chaguro* is a food supplement, made from *chayote* and tuna fish that is useful in specific plan diets for patients with diabetes. In the elaboration of *chaguro*, different proportions of tuna fish and *S. edule* were tested. Results revealed that the combination of 75% of dry tuna fish mixed with 25% of *S. edule* is useful to induce lower blood glucose and improve the lipid profile in DM and dyslipidemia [137].

The nutritional content of *S. edule* revealed great amounts of vitamin C, starch, proteins, peroxidases, and an interesting mineral content that includes metals such as iron, manganese, zinc, and calcium. In contrast, the sugar and carbohydrate content were detected in a notoriously low amount. Then, based on the mineral and carbohydrate content present in *chayote*, as well as flavonoids, *chayote* could be considered for use in the prevention of chronic diseases such as DM, cardiovascular diseases, and the metabolic syndrome [138].

### 3.7. Citrullus lanatus (Thunb.) Matsum. & Nakai

The most widespread common name worldwide for *Citrullus lanatus* is *watermelon*, but it is also called *sandía*, *tarbooz, acendría*, and *cooking melon* among others. The fruit of this *cucurbit* possesses approximately 93% water, and a large and globous shape with a sweet and pulpy flesh, the reasons for it being denominated *watermelon*. Africa has been considered as the center of origin of *watermelon*, where has been cultivated for more than 4000 years. At present, *watermelon* is a very popular cultivar through the world, where the main producers are China, Turkey, Greece, Italy, Spain, Mexico, and Japan [139,140]. The fruit of *C. lanatus* is consumed fresh and raw, but also cooked, especially in some parts of Asia. With regard to the traditional use of the watermelon, there are numerous and diverse reports for using as cooling, strengthening, aphrodisiac, expectorant, diuretic, blood purifier, to cure itches, urinary tract infections, and kidney stones. The rind of the fruit is recommended for the treatment of diabetes [126,139,141]. The pharmacological properties of *watermelon* have been identified as antibacterial, antifungal, antiulcer, anti-inflammatory, anti-prostatic hyperplasia, anti-atherosclerotic, gastroprotective, analgesic, hepatoprotective, antioxidant and antidiabetic [139,141,142]. Among the main phytochemicals detected in *watermelon* are carotenoids, which represent from 31% (yellow pulp cultivar) to 99% (red pulp cultivar) of the percentage of total carotenoids. It has been considered that *watermelon* is one of the best sources of lycopene, after tomato, but also the carotenoids vary depending on the variety of color of the *watermelon*, for example, red (lycopene and beta carotene), yellow (neoxanthin, violaxanthin, and luteoxanthin), orange (beta carotene, prolycopene, phytonene, and carotene), and white (carotene). Other phytochemicals reported in watermelon comprise phenolic compounds, vitamins, aminoacids, flavonoids, and alkaloids [139,143]. On the other hand, *watermelon* has also been recognized as a source of L-citrulline, a neutral, non-essential amino-acid, precursor of L-arginine in mammals, and an important component of the cycle of the urea in the liver and kidneys. L-citrulline is involved in the production of endogenous nitroxide (NO), which is considered essential for regulating vasodilatation, immune responses, neurotransmission, and the adhesion of platelets and leukocytes. Thus, in recent years, the potential benefits of supplementing L-citrulline have been explored in cardiometabolic areas that include skeletal muscle and adipose tissue metabolism [142,144].

The lycopene present in the pulp of the fruit of *Citrullus lanatus* was investigated for its possible antidiabetic properties in an in vitro assay, where it exerted the inhibition of the α-amylase and lipase enzymes, in addition to its high content of L-citrulline [144]. In a study that included extracts prepared from different organs from the same plant (seed, flesh, rind, and leaves), the aqueous extract of the leaves of *C. lanatus* was able to inhibit α-glucosidase, and it also exhibited higher antioxidant capacities. Some of the molecules identified in the leaf extract were curcumenol, curcubitacin E, citrulline, 6-gingerol, citric acid, ascorbic acid, leucine, arginine, palmitic acid, arjunolic acid, naringenin 5,7-dimethyl ether 4′-O-xylosyl-(1->4)-arabinoside, 4′-apo-beta, psi-caroten-4′-al, caffeic acid 3-glucoside, luteolin 7-rhamnosyl (1->6) galactoside, and apigenin 7-(4″,6″-diacetylalloside)-4′-alloside. These results permit proposing that watermelon could potentially be useful in the treatment of diabetes, but not only employing the fruit, but also the raw leaves [145]. Other work, exhibited that the administration of the juice of the watermelon to 40 Wistar rats and, was able to inhibit the enzymes α-amylase and α-glucosidase in a dose-dependent manner. The administration of different doses of *watermelon* juice reduced the fasting blood glucose level, serum lipid profile, glucose-6-phosphatase, lipid peroxidation, and anti-inflammatory activities in Alloxan-induced diabetic rats. These results revealed that the juice of the *watermelon* may be useful in the treatment of DM via multiple pathways, and could also be helpful in metabolic complications associated with DM [146]. Additionally, other investigations revealed that the ethanolic extract from the seeds of *watermelon* was not toxic when administered orally in rats in doses of 250, 500, 1000, and 2000 mg/kg for 28 days, in that no signs of toxicity, behavioral changes, or mortality were observed in the treated animals. Thus, the consumption of the entire pulp of the *watermelon* could be considered as safe [147]. The possible use of *watermelon* in the treatment of DM and in some related comorbidity diseases were explored in an in vivo study, where the extracts of the leaves of *C. lanatus* were administered in doses of 200 and 400 mg/kg to obese and diabetic-induced rats. The histological analysis demonstrated that the extracts of *watermelon* were able to attenuate the biochemical parameters and structural changes in the kidneys and liver of diabetic animals, indicating that the extract may be useful in complications associated with diabetes [148]. A different study showed that dietary supplementation with *watermelon* and L-citrulline reduced blood pressure in trials in humans probably plays an important role in the metabolism of lipids and in the control of satiety, consequently, *watermelon* could be useful in cardiac disease associated with diabetes [149].

### 3.8. Cucumis sativus L.

*C*. *sativus* known as *cucumber* is a Cucurbitaceae from India but it is currently widespread around the world. The fruit has been an essential part of the Mediterranean diet for centuries, it is mainly eaten fresh in salads or fermented. The pulp has been utilized for cleansing of the skin traditionally and today, *cucumber* extracts are employed in the cosmetic industry for many products [150]. Concerning its medicinal uses, *cucumber* has been reported as a laxative, as astringent, an anthelminthic, and an antipyretic. Other reported uses are in the treatment of hepatitis, bronchitis, asthma, diarrhea, and leprosy [151]. Notoriously, these traditional uses are mostly reports from Asia and Europe, but it is widely used in the entire Mexican territory for the treatment of DM [152].

The fruit possess antibacterial, antitumoral, antifungal, hepatoprotective, gastroprotective, ultraviolet protectant, wound-healing, and hypoglycemic activities [153]. This latter activity has been the object of recent studies, for example, the oral administration of 500 mg/kg of methanolic extract of the fruit caused a significant decrease in the fasting blood glucose concentration of Alloxan-induced diabetic rats, exhibiting a similar effect to that of Glibenclamide [154]. The ethanolic extract of the fruit of *C. sativus* demonstrated antihyperglycemic activity and also gave rise to the reduction in elevated lipid profiles in Alloxan-induced diabetic rats. It was notorious that these effects were more pronounced than those exhibited by other species of Cucurbitaceae, such as *Lagenaria siceraria* and *Cucurbita maxima* [155]. In an in vivo model, the anti-hyperglycemic effect of the decoction of *C. sativus* by the subcutaneous glucose tolerance in rabbits was determined. *C. sativus* was able to reduce the hyperglycemic peak significantly and was considered by the authors as recommendable for the prevention and treatment of DM [33]. Thus, the existing literature reveals the hypoglycemic effect of *C. sativus* extracts and their potential use in the treatment of DM. However, and to the best of our knowledge, none of the active molecule content in the extracts that have demonstrated this hypoglycemic activity has been isolated and identified by bio-guided studies of *C. sativus*.

On the other hand, general phytochemical screenings carried out on the leaf and fruit extracts of *C. sativus* revealed the presence of alkaloids, glycosides, steroids, saponins, flavonoids, and tannins [91]. Other investigations reported the presence of the triterpenoids cucurbitacins, which are characteristic of the Cucurbitaceae family in the fruits of *C. sativus*, such as the cucurbitancins A, B, C, D, E, and I, which are well known to confer the bitterness and toxicity found [156]. A comparative study of three different cultivars of Greek *C. sativus* showed the presence of 21 essential oils in which the majority compounds exhibited some variation among samples; however these main compounds comprised Z-6-nonenol (61.54%), E-2-nonenal (6.98%), E,Z-2,6-nonadienal (47.08%), E-2-nonenal (17.39%), Z-3-nonenol (14.79%), 3-nonenal (7.32%), pentadecanal (43.47%), 9,12,15-octadecatrienal (14.52%), and 9,17-octadecadienal (12.33%) [157]. A study focused on the interrelationships of the volatile constituents of *cucurbits* showed the presence of the 1-nonanol, trans-2-nonen-1-ol, cis-3-nonen-1-ol, cis-6-nonen-1-ol, trans, cis-2,6-nonadien-l-o1, cis, cis-3,6-nonadien-l-o1, cis-6-nonena1, cis-3-nonenal, and cis, cis-3,6-nonadienal in the fruit of *C. sativus* [158]. Other studies of the lipid composition of the fruit of cucumber with importance in the industry were focused on total lipids, neutral lipids, glycolipids, and phospholipids, among which palmitic and linolenic acids were the predominant fatty acids, followed by the lauric, myristic, stearic, oleic, imolenic, tricosanoic, tricosenoic, lignoceric, and nervonic acids [159].

Some other reports focused on different organs of the *C. sativus*, such the flowers, from which mainly flavonoids were isolated such as kaempferol 3-*O*-rhamnoside, and 3-*O*-glycosides of kaempferol, quercetin, and isorhamnetin, while C-glycosides were found in the leaves, including: isovitexin 2″-*O*-glucoside, isovitexin, isoorientin, 4′-X-*O*-diglucosides of isovitexin, and swertiajaponin [160].

Interestingly, and even when there is abundant literature on the phytochemicals of the *cucumber* from specimens around the world, and to the best to our knowledge, none of these phytochemical screenings considered biological activity (bio-guided) and even more so, they did not focus on hypoglycemic activity.

### 3.9. Cucumis melo L.

*Cucumis melo* is one of the fruits most consumed worldwide due to its delicious and juicy taste. It is also known as *cantaloupe*, *Musk melon*, *sweet melon*, *round melon*, and *melón*. In recent years, *C. melo* has become widely known because of its increasingly nutritive and medicinal functions [161,162]. Its chemical content, mainly fatty acids, polyphenols, and carotenoids have been recorded as possessing a broad range of beneficial characteristics that can also enhance health. Notoriously, the main source of these molecules is the mesocarp, which became the most studied part of the *melón*, compared with the skin and the seeds [163]. Some of the most abundant phenolic compounds detected in *C. melo* were reported in its seeds by Quian et al. [164], in which naringenin-7-O-glycoside, gallic acid, and vanillic acid were identified, while in the seed oils, γ-tocopherol, showed the highest concentration above that of α-tocopherol, β-tocopherol, and γ-tocotrienol. Flavonoids such as ß-carotene, lentin, xanthin, and cryptoxanthin were also reported.

The pharmacological properties of *C. melo* have been studied in recent decades and anti-inflammatory, analgesic, anticancer, antioxidant, anthelminthic, antimicrobial, antidiabetic, diuretic, and hepatoprotective effects were found [165]. Even traditional medicine has described the use of the fruit and the roots, the fruit is the most utilized part in the treatment of flatulence, obesity, fever, leprosy, bronchitis, anemia, constipation, hypertension, and diabetes [166,167].

Regarding the antidiabetic properties of *melón*, it is known that the gut microbiota plays an important role in the production of the metabolic endotoxins that can potentiate or inhibit insulin resistance. The administration of *C. melo* in obese mice reduces the inflammation induced by endotoxins of the bacterial flora, in addition to activating the function of insulin [168]. On the other hand, the potential use of the juice of *C. melo* has been investigated in the management of T2DM with cardioprotective effects in arteriosclerosis [169]. Interestingly, because *melón* is a commercial crop, there are varieties that have exerted antidiabetic activity, and these have been employed in traditional medicine around the world (Table 4), however, and to the best to our knowledge, the identification of the compounds responsible for the antidiabetic activities remain unknown.

## 4. Discussion

The persistent increase in the number of people worldwide who are diagnosed with DM, was estimated at 10.5% (536.6 million individuals), which has led to increasing novel and effective investigations that seek to offer treatments in order to contribute in a better control of DM, its associated complications, the patients’ quality of life, and, of course, to alleviate the global burden of DM in health sectors [176].

Consistently elevated blood glucose levels have been one of the major concerns for the control of patients with DM, numerous investigations have focused on the hypoglycemic effect. Nonetheless, in recent decades, the importance of the correlation among hyperglycemia, obesity, oxidative stress, and inflammation, especially during the development and progression of T2DM, has been established [177,178]. Obesity produces a state of chronic inflammation, and the oxidative stress is involved in the insulin resistance. The imbalance between the production of free radicals and their reduction by antioxidant mechanisms provoke insulin resistance and vascular complications [179,180]. Some substances, such as GSH and MDA are altered in patients with T2DM: GSH decreased and MDA increased. Therefore, MDA, GSH, and the redox potential determined by the ratio of GSH/GSSH (oxidized glutathione) are used as indicators of oxidative stress. Low concentrations of GSH and high levels of GSSG have been found in patients with T2DM and diabetic experimental animals as a result of oxidative stress [47]. In turn, oxidative stress activates inflammation pathways, with the consequent production of proinflammatory cytokines such as TNF-α, IL-6, IFN-γ, and resistin [181,182]. In patients with T2DM, an increase in the levels of inflammatory markers is observed [183]. Accordingly, a number of investigations have been exploring not only the hypoglycemic effect, but also the antioxidant and anti-inflammatory properties of T2DM in the area of modern drug discovery, especially in the field of edible plants, where this latter approach has become relevant due the additional impact that diets-based-on-vegetables have in T2DM patients. One of the most prominent plant families with all of these characteristics, as mentioned previously, is the Cucurbitaceae family [51].

Although the relevance of the species belonging to the Cucurbitaceae family in the empirical treatment of DM mainly through the hypoglycemic effect has been recognized, the isolation and identification of the active molecules responsible for this action remain [184]. Probably the most frequently used Cucurbitaceae in current traditional Mexican medicine for the treatment of DM, *I. sonorae*, possesses abundant cucurbitacin’s content in their roots. Because cucurbitacins comprise a very structurally diverse group of triterpenes that characterize the Cucurbitaceae family (but they could also be found in other families of plants) their pharmacological activity is varied. Hence, even though cucurbitacin-type triterpenes-Kinoin, which had been described, to our knowledge, for the first time in *wereke*, and characterizing this species, the hypoglycemic effects observed in in vitro and in vivo assays were attributed to the presence of monoglycerides and fatty acids localized in the roots [118]. Additionally, it was also observed that the antidiabetic properties of *wereke* may be related with the presence of flavonoids, and with high amounts of gallic acid [121]. In this manner, even with the presence of cucurbitacins, the antidiabetic properties observed in *wereke* were not clearly associated with these. Moreover, the cucurbitacins of *wereke* showed potent anticancer activity, and at present, a number of related investigations are in progress [111,112,113]. In a similar situation, the cucurbitacins present in *S. edule* are predominantly cucurbitancins B, E, P, and L, but pharmacological studies have described the flavonoids as the most relevant phytochemicals for hypoglycemic effects [134].

On the other hand, species from the genus *Cucurbita* have been the object-of-study in recent years, in that their chemical and nutritional contents have exhibited a wide spectrum of pharmacological activities. The chemical content in the species of this genus comprises carotenoids, tocopherols, phenols, terpenoids, saponins, sterols, fatty acids, carbohydrates, and polysaccharides [51]. However, the carbohydrate and polysaccharide content appears to be crucial for hypoglycemic effects and other related antidiabetic processes such as antioxidant and anti-inflammatory, in the species *C. ficifolia*, *C. maxima*, *C. moschata*, and *C. pepo* which are used in traditional Mexican medicine for the treatment of DM. Active fractions with antidiabetic properties identified in these species, were characterized with by a high content of polysaccharides from the aqueous extracts, while the antidiabetic activities also comprise pancreatic-cell protection [41,67,87,102,103].

It is interesting to note that in the case of *C. lanatus* and *C. melo*, the use in traditional medicine for the treatment of T2DM is notoriously more frequent in Mexico than in Asia, where both regions are found to be places of the commercial production of *watermelon* and *melón*. In the case of *C. lanatus*, the antidiabetic properties observed correlated with the presence of high amounts of lycopene and L-citrulline [144] in the fruits, while for the case of *C. melo*, the antidiabetic activity was associated with the presence of rutin, quercetin, gallic acid, linoleic acid, oleic acid, and unsaturated fatty acids [171,175]. As could be perceived in both cases, information on the antidiabetic effects and the active molecules responsible of this activity, are still scarce. Nonetheless, in the present work, from the total of selected species of Cucurbitaceae that are used in traditional Mexican medicine in the treatment of DM, *C. sativus* appears to be the least studied, in that very few reports demonstrated the hypoglycemic effects of *C. sativus* extracts [33], and, to best of our knowledge, none of these studies focused on the identification of the bioactive molecules. In contrast, a great number of studies that revealed the chemical composition of the essential oils of *C. sativus* are present in the literature, showing that the industrial application of the oils of *C. sativus* has probably been driving this approach.

In addition to the phytochemical content, because the Cucurbitaceae family has been recognized as one of the most widely used traditional antidiabetic family of plants worldwide, a crucial aspect in terms of continuing the research of potential new agents that could be useful in the treatment of DM, is the toxicity of the antidiabetic species. Although Marles and Farnsworth [185] reported that toxicity has been detected in the majority of the hypoglycemic species of Cucurbitaceae, in the present work, the selected species of Cucurbitaceae that are employed in Mexico to the treatment of DM are mostly edible and considered as safe, according to the literature. For the case of *C. ficifolia*, an acute toxicological study revealed that when the juice of immature fruits was administered to diabetic patients in a dose of 100 mg/kg, no toxicity effects were observed. Nonetheless, in an experimental model, deaths of animals were recorded in a dose of 625 mg/kg, but the dose employed in patients was evidently smaller than that used in animals. Despite this, these data revealed that the juice of *C. ficifolia* does not cause toxic effects in patients, more studies concerning the sub-acute administration of the juice are required [186]. The seeds of *C. maxima* were evaluated in an acute and subacute toxicological study. The lethal dose (DL_50_) of the hydroalcoholic extract of *C. maxima* was determined as higher than 5000 mg/kg when it was orally administered. Thus, the hydroalcoholic extract from the seeds of *C. maxima* can be considered as safe [187]. The ethyl acetate and methanolic extract of *C. pepo* did not show any toxicity in mice when administered in 2000 mg/kg doses for 14 days [188]. Non-toxicity signs were recorded after the oral administration of the ethanolic extract of the seeds of *C. lanatus* in Wistar rats at doses of 250, 500, 1000, and 2000 mg/kg; moreover, protection to different body organs was observed at under the 1000 mg/kg doses [147]. Recent studies suggested that the extracts of the seeds of *C. melo, C. lanatus, C sativus*, and *C. maxima* are not only safe but that they can also be effectively used in chronic pain conditions due the analgesic and anti-inflammatory properties shown [62,189]. Perhaps the highest dose evaluated experimentally in order to determine the toxicity effect on the species of Cucurbitaceae, is the dose of 5000 mg/kg, which was administered to mice over 7 days by means of the methanolic extract of *S. edule*. The absence of signs of toxicity suggested that *chayote* can be considered as safe, without any harmful side effects [190]. Nonetheless, it is one of the species arousing the most interest considered in the present work, since it is probably the most used plant in traditional Mexican medicine to the treatment of DM, which, despite the potent hypoglycemic effect exerted by the extracts prepared with the roots and the possible role of *wereke* in the prevention of diseases correlated with diabetes such as obesity, dyslipidemia, and hyperglycaemia, the administration of high doses of the extract (>1000 mg/kg) to diabetic rats may induce toxicity [120].

Through the previously mentioned information and because DM is a chronic disease, where the pharmacological treatment is managed over the lifetime of the patients, chronic-toxicity studies of the selected species of the Cucurbitaceae family considered in the present work must be exhaustively conducted in future investigations.

Another interesting aspect that we wish to highlight about the selected species of Cucurbitaceae that are included in the present work is the fact that the majority of these *cucurbits* are edible. These characteristic results are relevant because, in recent decades, a vegetables-based diet has shown to be central in diabetic patients for the control of DM due to the replacement of animal-derived lipids and proteins that directly impact the glucose homeostasis. Particularly, the majority of the Cucurbitaceae species are considered a good source of protein, and dietary fiber, which is important for the regulation of cholesterol, improvements of the insulinemic response in providing benefits for the intestinal function [13]. In recent years, in Mexico, efforts have been made to study edible plants that could contribute to the approach of a novel diet-therapeutic alternative to prevent chronic diseases such as DM. Then, an evaluation of the nutritional value, glycemic index (GI), and the total phenol and flavonoid content, as well as antioxidant and antidiabetic activity (inhibition of α-amylase and α-glucosidase) of plants used by the Maya population in Mexico was conducted. The results showed that *C. moschata*, among other plant species from different botanical families, possess antidiabetic properties that allow it to be incorporated as a food-diet in the treatment and prevention of DM [191]. Due the important role of Cucurbitaceae in the treatment of DM, a crucial study was carried out with patients with T2DM. In that study, diet was based on the consumption of the following six species of Cucurbitaceae for 2 months: *C. sativus*, *C. pepo*, *C. moschata*, *Lagenaria siceraria*, *C. lanatus*, and *C. melo*. Study results showed that the diet based on Cucurbitaceae was able to control the levels of blood sugar when employing the fasting blood sugar and glycated hemoglobin for measurements. Conveniently, the diet based on *cucurbits* was low in carbohydrates and lipids but rich in minerals and vitamins, according to the daily nutrimental requirements. However, certain other essentials need to be taken as supplements. Thus, this study confirms that the Cucurbitaceae family species could be useful in clinical research for the treatment of DM [27]. Interestingly, five of the six species comprised in that study are also included in the present work. Thus, the selected species of Cucurbitaceae employed in traditional Mexican medicine for the treatment of DM represent a potential source of agents for the treatment of DM from an integral perspective, in which the antidiabetic properties combined hypoglycemic, antioxidant, anti-inflammatory, and anti-obesity effects, and also for the control of dyslipidemia. Even more so, the Cucurbitaceae species considered in the present work possess the pharmacological potential to be developed as new antidiabetic agents, but also to be included as essential components of diets for the control of DM in patients with the disease. Finally, since the plant species treated in this manuscript that belong to the Cucurbitaceae family are used for the treatment of DM in Mexico are considered as safe, some future aspects to consider for research in this topic comprise the identification of the bioactive compounds in fractions or extracts that could provide fundamental information for the standardization of future antidiabetic phytomedicines, particularly, those bioactives that could possess multi-antidiabetic activities. On the other hand, another interesting aspect to explore in future research is the prevention or delay the complications associated with DM, and in which, the bioactives against DM present in Cucurbitaceae could also be beneficial. Thus, the interactions among the antidiabetic compounds present in Cucurbitaceae and the myokines that regulate the beneficial effects of the practice of exercise in patients with DM, may appear as the subject of prospective studies that could impact in the deterioration of organs caused by DM disease. In this manner, the Cucurbitaceae family represents a source of compounds that are perhaps useful in the DM therapy through different pathways.

## 5. Conclusions

The complexity of the DM disease and the increasing number of people who suffer from DM worldwide clamor for novel and effective treatments for its control. The use of plant-based traditional medicine for the treatment of DM is an extensive practice in Mexico and other countries where DM is a serious health problem. In the present work, the most used plants in the treatment of DM in Mexico that belong to the Cucurbitaceae family were reviewed with regard to their antidiabetic properties. From among the selected species, with the exception of *I. sonorae*, the remainder of the selected species are edible and are also considered as part of the diet recommended to diabetic patients in order to control DM. The antidiabetic effects shown by the selected Cucurbitaceae species comprise hypoglycemic, antioxidant and anti-inflammatory activities, anti-obesity and protective effects on diverse organs and cells, as well as in the control of dyslipidemias. Hence, the Cucurbitaceae species selected in the present work encourage future and expeditious investigations that would be able to make effective antidiabetic agents available that could contribute to the control of DM and perhaps establish the bases for novel clinical treatments.

## Figures and Tables

**Table 1:** Evaluation of the hypoglycemic, antioxidant and anti-inflammatory effects of the molecules isolated from different extracts of the fruit of *C. ficifolia*.

Extract	Molecules	Antidiabetic Evaluation	Results	Reference
Methanolic (70%)	D-*chiro*-inositol (DCI) (2.9 ± 0.2 mg/g), *myo*-inositol (7.8 ± mg/g), fagopyritols (33.6 ± 3.9 mg/g), and sucrose (126.8 ± 9.1 mg/g)	Antihyperglycemic effect of extract and synthetic DCI in Streptozotocin diabetic rats	↓ Level blood glucose ↑ Level hepatic glycogen ↑ Total hemoglobin ↑ Plasma insulin	[41]
Aqueous	DCI (3.3 mg/g)	Antioxidant and anti-inflammatory activity of extract and synthetic DCI in adipocytes	Extract and synthetic DCI: ↓ Oxidative stress ↑ Glutathione peroxidase activity DCI: ↓ TNF-α ↑ IL-6 ↓ Resistin	[46]
Aqueous precipitate extract	DCI (3.3 mg/g)	Antioxidant and anti-inflammatory activity of the extract in Streptozotocin-diabetic mice	↑ GSH ↓ MDA ↓ TNF-α ↑ IL-6 ↑ IFN-γ ↑ IL-10	[47]
Aqueous	*p*-Coumaric acid, *p*-hydroxybenzoic acid, salicin, stigmast-7,2,2-dien-3-ol, and stigmast-7-en-3-ol	Hypoglycemic effect of aqueous extract, glycogen liver quantification and histological analysis	↑ Liver glycogen accumulation ↑ Glycogen synthase ↓ Glycogen phosphorylase	[48]

**Table 2:** Antidiabetic activity of extracts prepared with different plant organs of *C. moschata*.

Part of the Plant Used/Extract	Antidiabetic Evaluation	Molecules Identified	Results	Reference
Fruit/Aqueous	Inhibition α-glucosidase	Galactose 86.4%, and glucose13.6%	Aqueous extract inhibited α-glucosidase enzyme in 97.4% at 0.7–0.9 mg/mL	[83]
Fruit/Ethanolic (95%)	Hypoglycemic activity on Streptozotocin induced diabetic mice	QGMG2 and QGMG3	QGMG2 and QGMG3 showed stronger blood glucose-lowering activity similar to Metformin	[81]
Fruit/Ethanolic (95%)	Hypoglycemic activity on Alloxan induced diabetic mice	Glucose, galactose, arabinose and rhamnose	Ethanolic extract fraction significantly reduced blood glucose levels in the diabetic mice	[84]
Fruit/Water-soluble polysaccharide fraction	Hypoglycemic activity on Alloxan induced diabetic rabbits	Glucose, galactose, arabinose, rhamnose, and hexuronic acid	Water-soluble polysaccharide fraction improved BG, TC, and HbA1c levels and stimulated β-cell proliferation	[85]
Steam/Methanolic	Hypoglycemic activity on Streptozotocin induced diabetic mice and molecular mechanisms	Loliolide, 2-hydroxybenzoic acid, 4-hydroxycinnamic acid, ferulic acid, (+)-(1*R*,2*S*,5*R*,6*S*)-2,6-di(4′-hydroxyphenyl)-3,7 dioxabicyclo [3.3.0]octane, pinoresinol, 4-ketopinoresinol, syringaresinol, (22*E*,24*R*)-24-methyl-6*β*-methoxy-5*α*-cholesta-7,22-diene- 3*β*,5-diol, and 3*β*-hydroxy-(22*E*,24*R*)-ergosta-5,8,22-trien-7-one	Steam extract showed hypoglycemic effect. Compounds (22*E*,24*R*)-24-methyl-6*β*-methoxy-5*α*-cholesta-7,22-diene- 3*β*,5-diol, and 3*β*-hydroxy-(22*E*,24*R*)-ergosta-5,8,22-trien-7-one: glucose uptake ↑ Compounds ferulic acid, syringaresinol, and (22*E*,24*R*)-24-methyl-6*β*-methoxy-5*α*-cholesta-7,22-diene- 3*β*,5-diol act as insulin sensitizers.	[86]
Seed and flesh/Ethanolic extract	Hypoglycemic activity on Streptozotocin induced diabetic mice		BG ↓	[87]

**Table 3:** Compounds identified by GC-MS from the culture of calluses of *I. sonorae*.

No.	Compound
1	Pentanamide
2	4-Methyl-3-penten-2-ol
3	4-Hexen-3-one
4	3-Hexen-2-one
5	3-Methyl-2-cyclopentanone
6	3-Methyl-hexane
7	2-Methyl-pentan-2,3-diol
8	4-Methyl-pentan-1,4-diol
9	2-Methyl-2-pentenol
10	3,5-Dimethyl-4-hydroxy-2-hexanone
11	2-Methyl-4-hydroxy-3-heptanone
12	1-Etoxy-3-methyll-2-pentene
13	2-Methylhepten-3-ol
14	3,4-Dimethylhexa-2-one
15	Decane
16	3-Methylencyclopentene
17	Undecane
18	Dodecane
19	Isopropyl 2-Butenate
20	5-Methyl-2carboxymethyl cyclopentanone
21	1-Cyclopentyl-5-methylhex-2-en-1-one
22	4,5-Dimethylnonane
23	Vynilbenzene
24	N,N-dibutylformamide
25	Methyl hexadecanoate
26	N-butyl ethanoate
27	1,3-Dimethylbenzene
28	1,2-Dimethylbenzene
29	1-Metyhl-4-isopropenylcyclohexene
30	Decanoic acid
31	n-Butyl 2-butenoate
32	1,3,5-Trimethylbenzene
33	2-Methyl-6-ethyldecane

Based on Estrada-Zúñiga et al. [123], and Morales and Siles [124].

**Table 4:** Antidiabetic activities of commercial varieties of *C. melo*.

Varieties	Country of Traditional Use	Part of the Plant/Extract	Antidiabetic Evaluation	Result	Reference
*C. melo* var. *momordica*	India	Fruit/Ethanolic extract and toluene fraction	Antihyperglycemic and antihyperlipidemic effect of the extract and fraction in Streptozotocin diabetic rats	Toluene fraction exhibited reduction in blood glucose levels (122 mg/dL); restored the levels of triglycerides (84.16 mg/dL), LDL 86.97 mg/dL), and VLDL (19.73 mg/dL)	[170]
*C. melo* var. *agrestis*	India	Leaves/Hydroalcoholic extract	Antihyperglycemic and antihyperlipidemic effect in Streptozotocin-nicotinamide induced diabetic rats	↓ Blood glucose level, ↓ HbA1c, ↓ total cholesterol, ↓ LDL, ↓ triglycerides levels, ↓ glycogen phosphorylase and ↓ glucose 6-phosphatase. Rutin, quercetin and gallic acid may be responsible for the activities	[171]
*C. melo* var. *conomon*	Japan	Fruit/Juice	10 patients with diabetes between the ages of 46 and 76 years drank the juices prepared with fruits that present different degrees of maturation	↓ Blood glucose level, The juice of the fully ripe fruit offered maximum benefits for patients with diabetes	[172]
*C. melo* var. *makuwa*	Korea, China	Seed/Ethanol and hexane extract	Inhibition of α-glucosidase and α-amylase enzymes	Extract exhibited strong inhibitory activities against α-glucosidase (35.3%) and α-amylase (61.8%)	[173]
*C. melo* var. *flexuosus*	Egypt	Leaves/Ethanolic extract	Neuroprotective effect in Streptozotocin diabetic rats	↓ Blood glucose level, ↓ brain tumor necrosis factor alpha and malondialdehyde. ↑ Dopamine, melatonin, brain vascularendothelial growth factor levels. Protective effect by the extract was observed to oxidative stress, inflammation, and neural death cell processes	[174]
*C. melo*	Lebanon	Seed/Hexanic extract	Inhibition of α-glucosidase and α-amylase enzymes	Extract inhibited both enzymes (α-glucosidase IC_50_ = 25.5 ± 1.9 μg/mL, and α-amylase IC_50_ = 191.6 ± 2.7 μg/mL). Unsaturated fatty acids, linoleic and oleic acids are responsible of activity observed	[175]

## Data Availability

Not applicable.
